# Asymmetric total synthesis of a putative sex pheromone component from the parasitoid wasp *Trichogramma turkestanica*

**DOI:** 10.3762/bjoc.10.71

**Published:** 2014-04-02

**Authors:** Danny Geerdink, Jeffrey Buter, Teris A van Beek, Adriaan J Minnaard

**Affiliations:** 1 Stratingh Institute for Chemistry, University of Groningen, Nijenborgh 7, 9747 AG, Groningen, The Netherlands; 2 Natural Products Chemistry Group, Laboratory of Organic Chemistry, Wageningen University, Dreijenplein 8, 6703 HB Wageningen, The Netherlands

**Keywords:** asymmetric catalysis, copper, deoxypropionates, natural products, sex pheromone, *Trichogramma turkestanica*

## Abstract

Virgin females of the parasitoid wasp *Trichogramma turkestanica* produce minute amounts of a sex pheromone, the identity of which has not been fully established. The enantioselective synthesis of a putative component of this pheromone, (6*S*,8*S*,10*S*)-4,6,8,10-tetramethyltrideca-2*E*,4*E*-dien-1-ol (**2**), is reported as a contribution to this identification. Catalytic asymmetric conjugate addition of methylmagnesium bromide and stereoselective Horner–Wadsworth–Emmons olefinations are used as the key steps, and **2** was obtained in 16 steps with an overall yield of 4.4%.

## Introduction

Communication by means of pheromones is common in many animal species. For instance, many insects secrete air-borne volatiles to attract a mate for generating offspring [1–2]. In 2005, one of us reported the isolation and partial characterization of the putative sex pheromone of *Trichogramma turkestanica*, a parasitoid wasp [3]. These wasps, belonging to the large Trichogrammatidae family, are minute in size (0.5 mm, 8 µg adult weight) as well as morphologically similar making a taxonomic characterization at the species level difficult [4]. A classification on the basis of sex pheromones might help in this respect.

**Figure 1:**
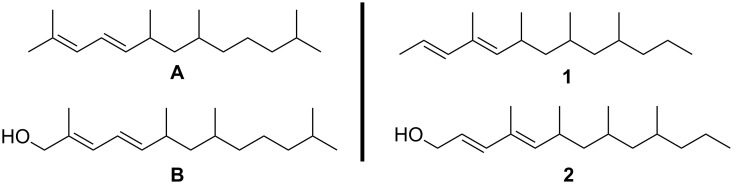
Originally proposed structures **A** and **B** and revised structures **1** and **2** of putative sex pheromone components of *Trichogramma turkestanica*.

Apart from the above reason and the scientific challenge to elucidate the identity of a complex pheromone at the low nanogram level, an additional reason to study these tiny wasps is their possible application in an environmentally friendly way of crop protection [5]. After copulation, the female wasp deposits the fertilized eggs inside the eggs of a host insect, which is used as a food source for the hatched wasps. Biological studies have revealed that only virgin females are able to trigger casting behavior in males, which has led to the assumption that virgin females of *Trichogramma turkestanica* produce a sex pheromone. Analysis of headspace volatiles of virgin females, collected via solid-phase microextraction, showed the presence of two sex specific components which were regarded to play an important role in this sex pheromone. After extensive analysis by mass spectrometry and derivatisation studies, the natural products were postulated to be 2,6,8,12-tetrametyltrideca-2,4-diene (**A**) and 2,6,8,12-tetramethyltrideca-2,4-dien-1-ol (**B**) (Figure 1) [3]. Stereochemical assignments were lacking. Subsequent synthesis of reference compounds led to revision of the structures, and as a result, the compounds were unambiguously identified to be (2*E*,4*E*)-*syn*,*syn*-4,6,8,10-tetramethyltrideca-2,4-diene (**1**) and (2*E*,4*E*)-*syn*,*syn*-4,6,8,10-tetramethyltrideca-2,4-dien-1-ol (**2**, Figure 1) [6]. The absolute configurations of the putative pheromone components, however, remained unknown. To solve this problem we undertook the stereoselective synthesis of **2**, thereby arbitrarily choosing the all-*S* configuration.

## Results and Discussion

**Scheme 1:**
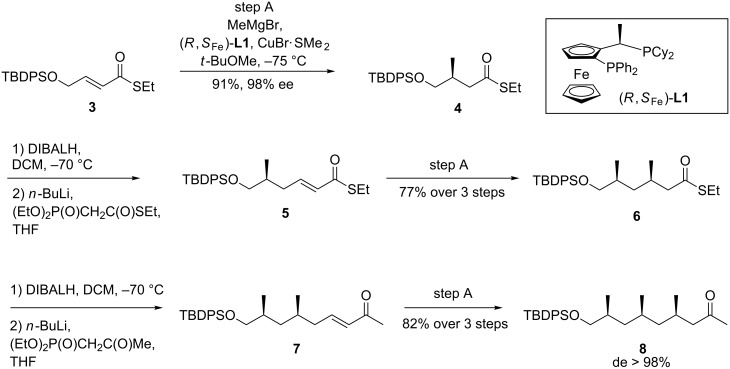
Application of the iterative conjugate addition protocol for the preparation of **8**.

Nowadays, a number of efficient strategies is available for the synthesis of deoxypropionates [7–15]. Our approach [16] is based on copper-catalyzed asymmetric conjugate addition of methylmagnesium bromide to α,β-unsaturated thioesters and has proven its versatility [17–19]. Arrays of up to eight methyl substituents have been constructed [20]. Starting from **3**, the first conjugate addition using *(R,S*_Fe_*)*-**L1** afforded **4**, as expected in high yield and excellent enantiomeric excess (Scheme 1). Reduction with DIBALH, followed by Horner–Wadsworth–Emmons olefination and again asymmetric conjugate addition gave **6** in 77% yield over three steps. To ultimately remove the carbonyl function, we introduced the third methyl ramification in α,β-unsaturated ketone **7**, preparation of which was straightforward [21]. Conjugate addition to **7** using the conditions from step A proved clearcut and gave **8** in 82% yield over three steps from **6**. Both the enantiomeric excess and the diastereomeric excess of **8** were excellent which is important as the response of insects to their pheromones can be highly sensitive to the stereoisomeric composition.

**Scheme 2:**
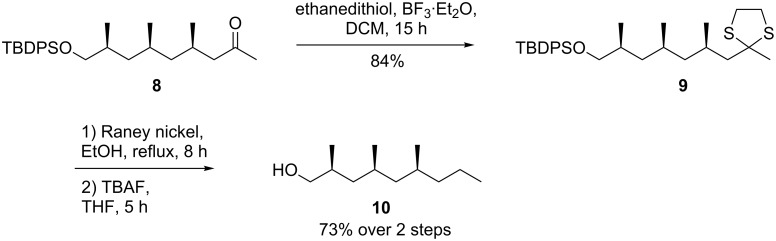
Deoxygenation and desilylation of **8**.

A number of procedures exist to reduce ketones to their corresponding methylene groups. Initial attempts focused on the formation and subsequent removal of the corresponding hydrazones. Regrettably, the Myers modification of the Wolf–Kishner reduction did not afford detectable amounts of **10** [22]. Also the modification of Caglioti proved only moderately successful with 30% isolated yield of **10** over two steps [23]. As an alternative deoxygenation procedure, we relied on the so-called Mozingo reduction. Although the procedure for dithiane formation has been reported to be catalytic in Lewis acid, we found a linear correlation between the amount of BF_3_·Et_2_O used and the conversion (Scheme 2) [24]. The use of 1.2 equiv of BF_3_·Et_2_O led to **9** in 84% isolated yield. Reduction of **9** with freshly prepared Raney nickel and subsequent desilylation afforded **10** over two steps in 73% yield.

**Scheme 3:**
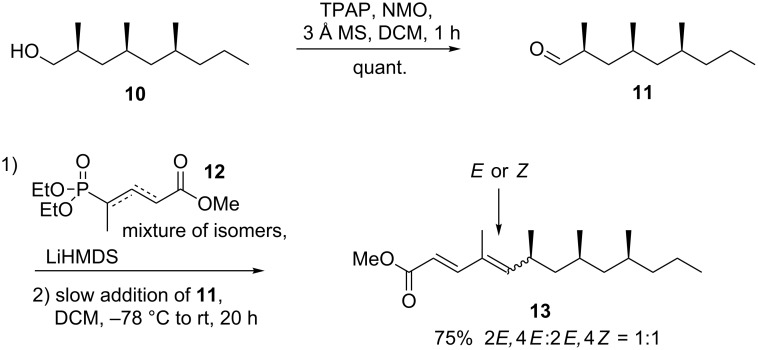
Vinylogous Horner–Wadsworth–Emmons olefination.

To introduce the desired *E,E*-diene present in **2** and **1**, we realized that the procedure reported by Markiewicz, comprising a vinylogous Horner–Wadsworth–Emmons olefination, offered in principle a very efficient procedure to obtain dienoate **13** (Scheme 3), and would leave only a single step in the synthesis of **2** [25]. Therefore, reagent **12** was prepared in two steps. After quantitative conversion of **10** into **11** using Ley–Griffith oxidation, its reaction with **12**, using LiHMDS as the base, afforded **13** in 75% yield, though as a 1:1 mixture of 2*E*,4*E*- and 2*E*,4*Z-*isomers. Moreover, the ^1^H NMR spectra indicated the presence of an impurity, which appeared as a multiplet between 5.00 and 5.45 ppm. Although minimal amounts of 2*E*,4*Z*-**13** could be isolated, 2*E*,4*E*-**13** was inseparable from this impurity. We decided to continue the synthesis with impure **13**, which was reduced with DIBALH at low temperature to give crude **1** in 96% yield. TLC analysis indicated the presence of two major, separable, products. Purification by flash-column chromatography over either silica gel or neutral aluminium oxide, however, led to rapid degradation of **2**. In the process, we were unable to isolate a pure sample of either isomer. This approach was ultimately abandoned, and only delivered the knowledge that **2** is unstable upon purification by column chromatography. This sensitivity to acid has been reported before for similar compounds [26], and can be explained by the readily formed dienyl cation.

We realized that, in order to obtain a pure sample of **2**, it was essential to avoid chromatographic purification after the final step. As the reduction of **13** with DIBALH is a clean reaction, affording essentially pure **2** after work-up, the preparation of pure **13** was highly desirable. A stepwise olefination approach was therefore considered.

**Scheme 4:**
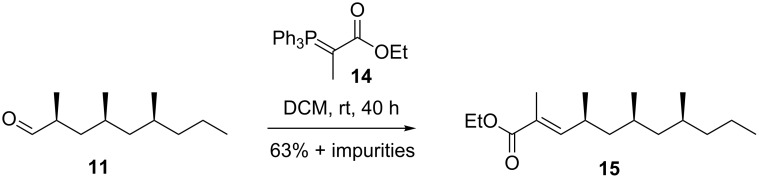
Synthesis of α,β-unsaturated ester **15** using a Wittig reaction.

Wittig reaction of aldehyde **11** with phosphorane **14** to give **15** was carried out first (Scheme 4) [27]. Next to ~60% of the desired product, around 40% of the mass balance consisted of an inseparable impurity. ^1^H NMR spectroscopy again showed a set of multiplets between 4.95–5.00 ppm. Although repeated recrystallization of commercial reagent **14** reduced the impurity to 6%, pure **15** could not be obtained, not even with freshly prepared **14**. Moreover, in none of the successive steps the impurity could be separated and therefore also this approach was abandoned. Although NMR and GC–MS analysis of the mixture gave no conclusive evidence for the nature of the impurity, double bond isomers of **15** could account for the observed ^1^H NMR signals.

**Scheme 5:**
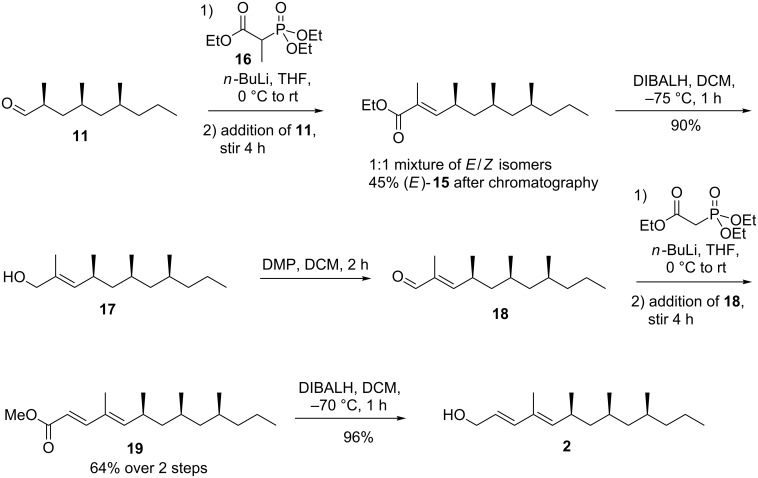
Completion of the synthesis of the putative sex pheromone **2**.

As the Wittig reaction of **11** did afford **15**, although with the simultaneous formation of an inseparable side-product, we were curious to see how the analogous Horner–Wadsworth–Emmons olefination towards **15** would perform. Thus, aldehyde **11** was freshly prepared and subjected to the conjugate base of **16** (Scheme 5). This olefination proved to be much faster, but afforded **15** as a 1:1 mixture of *E-* and *Z*-isomers as determined by ^1^H NMR spectroscopy. This is attributed to a destabilizing interaction of the α-methyl moiety of the HWE reagent in the transient four-centered intermediate, leading to a mixture of double bond isomers. The impurity previously observed as a result of the Wittig reaction was not present. In addition, the *E*- and *Z*-isomers could be separated using column chromatography affording pure *E*-**15** in 45% yield. Reduction of **15** was achieved using DIBALH, affording allylic alcohol **17** in 90% yield, which in turn was oxidized to aldehyde **18** using Dess–Martin periodinane. Given that the conversion of **18** into **19** using a Wittig reaction had proven to be sluggish, we switched again to a Horner–Wadsworth–Emmons olefination, expecting to observe high *E*-selectivity. Indeed, **19** was obtained in 64% yield over two steps, as the pure *E,E*-isomer. Reduction of pure **19** with DIBALH finally afforded **2** in 96% yield after work-up. The compound showed to be identical to the natural product and *rac*-**2** prepared in the accompanying paper [6] on the basis of two GC retention times (polar and nonpolar column) and mass spectrum.

## Conclusion

In summary, after two unsuccessful attempts, (*E,E,S,S,S*)*-***2** has been obtained in a linear sequence of 16 steps and 4.4% overall yield. As the absolute configuration of natural **2** is yet unknown, comparison of natural **2** with now available synthetic enantiopure and racemic **2** will be the next step. Separation of racemic **2** by chiral GC has up till now shown impossible, however. The preparation of deoxy analogue **1** is currently part of our investigations.

## Supporting Information

File 1 Detailed experimental procedures and spectral data of all new compounds.
