# Supplementary material for: Asymmetric total synthesis of a putative sex pheromone component from the parasitoid wasp Trichogramma turkestanica
Source: Beilstein J Org Chem. 2014 Apr 2;10:761–6. doi: 10.3762/bjoc.10.71 (PMC3999798; doi:10.3762/bjoc.10.71)
Supplement: File 1 — Detailed experimental procedures and spectral data of all new compounds. [file Beilstein_J_Org_Chem-10-761-s001.pdf]

Supporting Information

for

Asymmetric total synthesis of a putative sex pheromone component from the parasitoid wasp *Trichogramma turkestanica*

Danny Geerdink[1], Jeffrey Buter[1], Teris A. van Beek[2] and Adriaan J. Minnaard*[1]

Address: [1]Stratingh Institute for Chemistry, University of Groningen, Nijenborgh 7, 9747 AG, Groningen, The Netherlands and [2]Natural Products Chemistry Group, Laboratory of Organic Chemistry, Wageningen University, Dreijenplein 8, 6703 HB Wageningen, The Netherlands.

Email: Adriaan J. Minnaard* - a.j.minnaard@rug.nl

* Corresponding author

Detailed experimental procedures and spectral data of all new compounds

Table of contents

1. General remarks and procedures S2
2. Spectral data of all new compounds S3
3. NMR spectra of all new compounds S8

General remarks

All reactions were performed using oven or flame-dried glassware and dry solvents. Solvents were taken from an MBraun solvent purification system (SPS-800). All other reagents were purchased from Sigma Aldrich, Acros, TCI Europe, Alfa Aesar, Chempur or Fluorochem and used without any further purification unless noted otherwise. Grignard reagents were titrated using *s*-BuOH and catalytic amounts of 1,10-phenanthroline. Fatty acids **3** to **7** were prepared according to a previously reported route [1].

^{1}H and ^{13}C NMR spectra were recorded on a Varian AMX400 or a Varian 400-MR (400, 100.59 MHz, respectively) using $CDCl_3$ or CD_3OD as solvent, unless stated otherwise. Chemical shift values are reported in ppm with the solvent resonance as the internal standard ($CDCl_3$: δ 7.26 for ^{1}H, δ 77.0 for ^{13}C, CD_3OD: δ 3.31 for ^{1}H) or using TMS. Data are reported as follows: chemical shifts (δ), multiplicity (s = singlet, d = doublet, dd = double doublet, td = triple doublet, t = triplet, q = quartet, br = broad, m = multiplet), coupling constants *J* (Hz), and integration. High resolution mass spectra (HRMS) were recorded on a Thermo Scientific LTQ Orbitrap XL or on a AEI-MS-902 spectrometer.

Flash chromatography was performed using SiliCycle silica gel type SiliaFlash P60 (230–400 mesh) as obtained from Screening Devices or with automated column chromatography using a Reveleris flash purification system purchased from Grace Davison Discovery Sciences. TLC analysis was performed on Merck silica gel 60/Kieselguhr F254, 0.25 mm. Compounds were visualized using either Seebach's reagent (a mixture of phosphomolybdic acid (25 g), cerium(IV) sulfate (7.5 g), H_2O (500 mL) and H_2SO_4 (25 mL)) or a $KMnO_4$ stain (K_2CO_3 (40 g), $KMnO_4$ (6 g), H_2O (600 mL) and 10% NaOH (5 mL)).

General procedure for the preparation of Raney nickel (procedure A)

An aqueous solution of NaOH (6.4 M, 500 mL) was cooled with an ice/salt bath. To the cooled solution a nickel/aluminium alloy (Ni:Al = 50:50, 100 g) was added in small portions over 2 h. The temperature was never allowed to rise above 15 °C. After addition, the ice/salt bath was removed and the suspension was allowed to warm to rt. The water was decanted and an aqueous solution of NaOH (2.5 M, 200 mL) was added to the residue. Stirring was applied for 15 min, whereafter the suspension was allowed to settle. Decantation of the alkali solution was performed and the residue was washed with water. Washing and decantation was repeated until the washings were pH neutral. The Raney nickel residue was washed with three portions of EtOH (95%, 600 mL) and three times with absolute EtOH (600 mL). The Raney nickel was stored under absolute ethanol.

General procedure for the Ley–Griffith oxidation of primary alcohols (procedure B)

To a stirred solution of alcohol in dry DCM were added freshly activated 4 Å molecular sieves (200 mg), *N*-methylmorpholine *N*-oxide (NMO, 2.1 equiv) and tetrapropylammonium perruthenate (TPAP, 5 mol %). The solution was stirred for 1 h whereafter it was flushed over a pad of silica with pentane/Et_2O (1:1). The filtrate was concentrated under reduced pressure affording the corresponding aldehyde.

(4*R*,6*S*,8*S*)-9-((*tert*-Butyldiphenylsilyl)oxy)-4,6,8-trimethylnonan-2-one (8): [1]

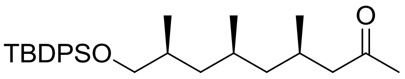

(R,S_{Fe})-Josiphos·CuBr complex **L1** (29.7 mg, 0.04 mmol, 1 mol %) was dissolved in *t*-BuOMe (25 mL) under a nitrogen atmosphere. The mixture was cooled to −80 °C and methylmagnesium bromide (1.60 mL, 4.80 mmol, 3 M in Et_2O, 1.2 equiv) was added dropwise over 15 min. After stirring for an additional 20 min, a solution of ketone **7** (1.69 g, 4.00 mmol, prepared according to a previously reported procedure) in *t*-BuOMe (6.8 mL) was added over 1.5 h using a syringe pump. The reaction mixture was stirred at −80 °C for 18 h, then quenched by addition of MeOH (25 mL) and allowed to warm to rt. Saturated aq NH_4Cl solution (35 mL) was then added. After phase separation and three extractions of the aqueous phase with Et_2O (120 mL), the combined organic phases were dried over Na_2SO_4, filtered, concentrated under reduced pressure and purified by flash chromatography (pentane/Et_2O 7:1) to afford **8** a colorless oil (1.21 g, 91% yield, *syn/anti* ratio by ^{1}H NMR = 98:2).

^{1}**H NMR** (400 MHz, $CDCl_3$) δ 7.73 – 7.54 (m, 4H), 7.54 – 7.31 (m, 6H), 3.51 (dd, *J* = 9.8, 5.1, 1H), 3.41 (dd, *J* = 9.8, 6.5, 1H), 2.47 – 2.28 (m, 1H), 2.18 – 1.98 (m, 5H), 1.81 – 1.64 (m, 1H), 1.51 – 1.41 (m, 1H), 1.41 – 1.33 (m, 1H), 1.19 – 1.11 (m, 1H), 1.10 – 1.05 (m, 1H), 1.06 (s, 9H), 0.98-0.88 (m, 1H), 0.93 (d, *J* = 6.6, 3H), 0.85 (d, *J* = 6.2, 3H), 0.82 (d, *J* = 6.4, 3H); ^{13}**C NMR** (101 MHz, $CDCl_3$) δ 209.15, 135.64, 135.61, 134.05, 134.02, 129.51, 127.57, 68.74, 50.85, 45.01, 41.20, 33.10, 30.43, 27.64, 26.90, 26.78, 20.75, 20.60, 19.32, 18.06; **HRMS**-(ESI+) for $C_{28}H_{43}O_2Si$ $[M + H]^+$ calculated 439.3027, found 439.3027.

***tert*-Butyldiphenyl(((2*S*,4*S*,6*R*)-2,4,6-trimethyl-7-(2-methyl-1,3-dithiolan-2-yl)heptyl)oxy)silane (9):**

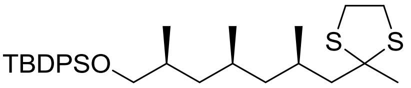

Ketone **8** (0.50 g, 1.3 mmol) and ethanedithiol (0.27 g, 2.9 mmol, 2.3 equiv) were dissolved in dry DCM (25 mL) and cooled with an ice/salt bath. To the cooled solution were added 4 Å molecular sieves and BF_3·OEt_2 (0.204 g, 1.44 mmol, 1.2 equiv). The resulting solution was allowed to warm to rt and was stirred 15 h under a nitrogen atmosphere. The reaction mixture was quenched with an aqueous solution of 5% NaOH (10 mL). After phase separation, the aqueous phase was extracted three times with DCM (75 mL). The organic phase was dried using Na_2SO_4, filtered and concentrated under reduced pressure. Flash chromatography was performed (pentane/Et_2O 25:1) to isolate **9** as a colorless oil (0.49 g, 84% yield).

^{1}**H NMR** (400 MHz, $CDCl_3$) δ 7.74 – 7.67 (m, 4H), 7.49 – 7.36 (m, 6H), 3.56 (dd, *J* = 9.8, 5.0, 1H), 3.46 (dd, *J* = 9.8, 6.5, 1H), 3.39 – 3.26 (m, 4H), 2.06 – 2.02 (m, 1H), 1.83 – 1.71 (m, 6H), 1.60 – 1.51 (m, 1H), 1.47 – 1.38 (m, 1H), 1.32 (m, 1H), 1.10 (s, 9H), 1.02 (d, *J* = 6.3, 3H), 0.98 (d, *J* = 6.7, 3H), 0.96 – 0.90 (m, 2H), 0.88 (d, *J* = 6.5, 3H); ^{13}**C NMR** (101 MHz, $CDCl_3$) δ 135.57, 135.56, 134.37, 134.33, 134.02, 130.17, 129.42, 127.83, 127.52, 68.70, 66.79, 51.78, 46.91, 41.09, 39.74, 39.03, 33.14, 33.02, 29.25, 27.66, 26.90, 25.97, 22.49, 20.97, 19.28, 18.16; **HRMS**: no successful mass analysis could be obtained due to extensive fragmentation. $[\alpha]_D$=-9.6 (c=0.90, $CHCl_3$)

***tert*-Butyldiphenyl(((2*S*,4*S*,6*S*)-2,4,6-trimethylnonyl)oxy)silane (9a):**

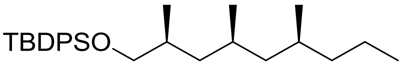

To a solution of **9** (447 mg, 0.87 mmol) in dry EtOH (25 mL) was added an excess of freshly prepared Raney nickel. The reaction was refluxed for 8 h, cooled down to rt, and filtered over a SiO_2 column (pentane/Et_2O 5:1). The filtrate was dried over Na_2SO_4 and concentrated under reduced pressure. Flash chromatography (pentane) afforded the title compound as a colorless oil (300 mg, 82% yield).

^{1}H NMR (400 MHz, $CDCl_3$) δ 7.93 – 7.65 (m, 4H), 7.65 – 7.38 (m, 6H), 3.61 (dd, *J* = 9.8, 5.0, 1H), 3.52 (dd, *J* = 9.7, 6.4, 1H), 1.83 (dt, *J* = 13.0, 6.6, 1H), 1.73 – 1.53 (m, 2H), 1.53 – 1.22 (m, 6H), 1.16 (s, 9H), 1.03 (d, *J* = 6.7, 3H), 0.98 – 0.93 (m, 5H), 0.92 (d, *J* = 3.6, 3H), 0.90 (d, *J* = 3.5, 3H); **^{13}C NMR** (101 MHz, $CDCl_3$) δ 135.95, 135.93, 134.74, 134.71, 134.42, 134.39, 130.54, 129.76, 128.19, 127.85, 69.10, 45.78, 41.76, 39.18, 33.51, 30.05, 27.93, 27.21, 26.30, 21.18, 20.84, 20.30, 19.63, 18.40, 14.77; **HRMS**-(ESI+) for $C_{28}H_{45}OSi$ $[M + H]^+$ calculated 425.3234 Da, found 425.3234 Da. $[\alpha]_D$=-7.2 (c=1.07, $CHCl_3$)

(2*S*,4*S*,6*S*)-2,4,6-Trimethylnonan-1-ol (10):

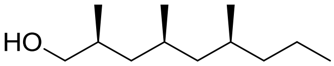

To a solution of **9a** (300 mg, 0.71 mmol) in dry THF (10 mL), was added TBAF (2.1 mL, 2.1 mmol, 3.0 equiv, 1 M solution in THF) under a nitrogen atmosphere. The resulting reaction mixture was stirred for 5 h and subsequently concentrated under reduced pressure. Flash chromatography (eluent pentane/Et_2O 5:1) was performed to obtain **10** as a colorless oil (117 mg, 89% yield). The product contained trace amounts of siloxane.

^{1}H NMR (400 MHz, $CDCl_3$) δ 3.51 (dd, *J* = 10.4, 4.9, 1H), 3.33 (dd, *J* = 10.3, 7.0, 1H), 1.80 – 1.64 (m, 2H), 1.60 – 1.43 (m, 2H), 1.39 – 1.11 (m, 6H), 0.91 (d, *J* = 6.6, 3H), 0.88 – 0.82 (m, 10H); **^{13}C NMR** (101 MHz, $CDCl_3$) δ 68.41, 45.36, 41.51, 39.03, 33.29, 29.94, 27.73, 21.11, 20.63, 20.14, 17.74, 14.61; **HRMS**-(ESI^-) for $C_{12}H_{25}O$ [M — H^+] calculated 185.1911 Da, found 185.1910 Da. $[\alpha]_D$=-13.6 (c=0.92, $CHCl_3$)

(2*E*,6*S*,8*S*,10*S*)-Methyl 4,6,8,10-tetramethyltrideca-2,4-dienoate (13):

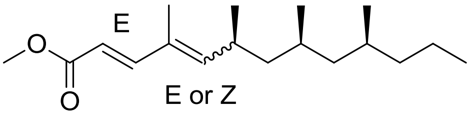

<u>*Procedure 1*</u>. The Ley–Griffith oxidation of alcohol **10** (120 mg, 0.64 mmol) to the corresponding aldehyde with TPAP (12 mg, 32 μmol, 5 mol %) and NMO (156 mg, 1.33 mmol, 2.0 equiv) was performed according to procedure B. The aldehyde was obtained as a colorless oil (119 mg, quantitative yield) and used without further purification. To a cooled solution of phosphonate **12** (234 mg, 0.94 mmol, 1.5 equiv) in dry THF (6 mL) at −78 ºC, lithium bis(trimethylsilyl)amide (LHMDS, 146 mg, 0.87 mmol, 1 M in THF, 1.4 equiv) was slowly added under a nitrogen atmosphere. The resulting solution was allowed to warm to rt for 30 min where after the solution was cooled to −78 °C. To the cooled solution, aldehyde **11** (115 mg, 0.62 mmol) in dry THF (5 mL) was added dropwise. The reaction mixture was allowed to warm to rt and was stirred for 20 h. The reaction was quenched with

a saturated aq solution of NH_4Cl (1.5 mL) and Et_2O (10 mL) was added. After phase separation, the aqueous phase was extracted with Et_2O (3 × 30 mL) and the combined organic layers were dried over Na_2SO_4, filtered and concentrated under reduced pressure. Flash chromatography was performed (pentane/ether 50:1) but the *E,E*- and *E,Z*-isomers could not be completely separated. A colorless oil was isolated (131 mg, 75% yield) with a 1:1 ratio of dienoate isomers (*E,E* / *E,Z*) as observed by ^{1}H-NMR spectroscopy.

<u>Procedure 2:</u>
The Ley–Griffith oxidation of allyl alcohol **17** (30 mg, 0.12 mmol) to the corresponding aldehyde with TPAP (2.3 mg, 6.0 µmol, 5 mol %) and NMO (28 mg, 0.24 mmol, 2.0 equiv) was performed according to procedure B. The α,β-unsaturated aldehyde **18** was obtained as a colorless oil (119 mg, quantitative yield) and used without further purification. **18** (29 mg, 0.113 mmol) was then dissolved in dry DCM (5 mL) and methyl (triphenylphosphoranylidene)acetate (47 mg, 0.14 mmol, 1.2 equiv) was added. The resulting solution was stirred for 17 h under a nitrogen atmosphere. The reaction did not result in full conversion, even not after stirring for 3 h with 0.2 equiv of additional phosphorane. The reaction mixture was concentrated under reduced pressure and flash chromatography (eluent pentane/ether 25:1) was performed. The obtained colorless oil proved to contain substantial amounts of α,β-unsaturated aldehyde **18**. The crude oil was redissolved in dry benzene (6 mL) and methyl (triphenylphosphoranylidene)acetate (47 mg, 0.14 mmol, 1.2 equiv) was added. The resulting solution was heated to 70 °C for 24 h under a nitrogen atmosphere. Incomplete conversion was observed and additional phosphorane (0.5 equiv) was added. Heating was continued for another 48 h. Again significant amounts of starting material were present in the reaction mixture. Heating was continued with addition of in total 1.5 equiv of phosphorane over 3 d. The reaction mixture was concentrated under reduced pressure and flash chromatography (pentane/Et_2O 50:1) was performed to obtain **19** (18 mg, 47%), containing approximately 6% of α,β-unsaturated aldehyde **18** as observed by ^{1}H NMR.

***(E,Z)*-19**: **^{1}H NMR** (400 MHz, $CDCl_3$) δ 7.76 (d, *J* = 15.6 Hz, 1H), 5.88 (d, *J* = 15.7 Hz, 1H), 5.46 (d, *J* = 9.7 Hz, 1H), 3.76 (s, 3H), 2.86 (dt, *J* = 10.1 Hz, 5.7 Hz, 1H), 1.84 (s, 3H), 1.62 – 0.65 (m, 22H); **^{13}C NMR** (101 MHz, $CDCl_3$) δ 167.98, 146.38, 141.65, 129.36, 117.60, 51.47, 45.60, 44.72, 39.34, 29.98, 29.59, 27.93, 22.04, 20.36, 20.03, 19.97 - 19.94, 14.38; **HRMS**-(ESI) for $C_{18}H_{33}O_2$ $[M + H]^+$ calculated 281.2475 Da, found 281.2436 Da.

***(E,E)*-19**: **^{1}H NMR** (400 MHz, $CDCl_3$) δ 7.31 (d, *J* = 15.7, 1H), 5.78 (d, *J* = 15.7, 1H), 5.63 (d, *J* = 9.6, 1H), 3.75 (s, 3H), 2.70 -2.59 (m, 1H), 1.78 (s, 3H), 1.50 – 1.43 (m, 1H), 1.43 – 1.17 (m, 6H), 1.16 – 1.10 (m, 1H), 1.09 – 0.99 (m, 2H), 0.96 (d, *J* = 6.6, 3H), 0.87 (t, *J* = 7.2, 3H), 0.81 (d, *J* = 5.4, 3H), 0.79 (d, *J* = 5.6, 3H); **^{13}C NMR** (101 MHz, $CDCl_3$) δ 167.64, 149.91, 148.63, 131.27, 115.48, 60.13, 45.69, 44.62, 39.33, 30.85, 29.62, 28.10, 21.22, 20.36, 20.04, 20.01, 14.39, 14.35, 12.34;

(4*S*,6*S*,8*S*,*E*)-Ethyl 2,4,6,8-tetramethylundec-2-enoate (15):

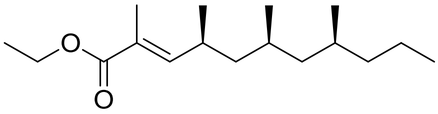

<u>Procedure 1.</u> To a stirred solution of aldehyde **11** (69 mg, 0.37 mmol) in DCM (15 mL) was added (carbethoxyethylidene)triphenylphosphorane **14** (244 mg, 0.67 mmol, 1.8 equiv). The resulting solution was stirred for 16 h under a nitrogen atmosphere, but the reaction did not proceed to full conversion. Additional **14** (0.5 equiv) was added, and the reaction was

extended for 24 h but full conversion was again not obtained. The resulting reaction mixture was concentrated under reduced pressure and flash chromatography (pentane/ether 25:1) was performed to afford the product as a colorless oil (64 mg, 63% yield). ^{1}H NMR analysis showed a multiplet ranging from 4.9 to 5.4 ppm which corresponded to approximately 6% of an unidentified side-product.

Note: Washing the (carbethoxyethylidene)triphenylphosphorane **14** with an aqueous solution of 10% Na_2CO_3 followed by re-crystallization from EtOAc showed to reduce the formation of the unidentified product (39% vs 6%).

Procedure 2. To a solution of **16** (1.8 equiv, 42 µL, 0.20 mmol) in THF (1 mL) at 0 ºC was added *n*-BuLi (1.4 equiv, 95 µL, 0.15 mmol, 1.6 M solution in hexanes). After 15 min, aldehyde **11** (20 mg, 0.11 mmol, dissolved in 0.2 mL THF) was added. The reaction was allowed to stir for 8 h, after which ^{1}H NMR indicated complete consumption of the starting material and showed the formation of a 1:1 E/Z-isomeric mixture. (*E*)-**15** (13 mg, 45%) was obtained after careful column chromatography (pentane/Et_2O 150:1).**^{1}H NMR** (400 MHz, $CDCl_3$) δ 6.53 – 6.47 (m, 1H), 4.25 – 4.09 (m, 2H), 2.69 – 2.54 (m, 1H), 1.85 (s, 3H), 1.52 – 1.43 (m, 1H), 1.38 – 1.15 (m, 9H), 1.21 (t, *J* = 7.0 Hz, 3H), 0.98 (d, *J* = 6.7 Hz, 3H), 0.91 – 0.86 (m, 6H), 0.84 – 0.80 (m, 3H); **^{13}C NMR** (101 MHz, $CDCl_3$, product from procedure 1) δ 168.38, 148.16, 126.18, 60.29, 45.55, 44.30, 39.28, 38.87, 30.84, 29.58, 28.07, 20.59, 20.41, 19.95, 14.35, 14.25, 12.44; **HRMS** no successful mass analysis could be obtained.

(4*S*,6*S*,8*S*,*E*)-2,4,6,8-Tetramethylundec-2-en-1-ol (17):

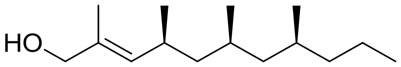

To a solution of **15** (15 mg, 0.056 mmol) in DCM (2 mL) at −75 ºC was added DIBALH (3 equiv, 0.17 mL, 1 M solution in DCM). After 1 h, TLC indicated complete conversion and the reaction was quenched with an aq saturated Rochelle salt (2 mL). The product was extracted with Et_2O (3 × 5 mL) and the combined organic layers were dried ($MgSO_4$), filtered and all volatiles were evaporated. **17** (11.4 mg, 90%) was obtained as a colorless oil, with traces of an unknown impurity, after filtration over a short silica plug (pentane/Et_2O 20:1).

^{1}H NMR (400 MHz, $CDCl_3$) δ 5.12 (d, *J* = 9.4 Hz, 1H), 3.98 (s, 2H), 3.45 (dd, *J* = 40.5 Hz, 5.9 Hz, 1H), 2.48 (m, 1H), 1.75 – 1.64 (m, 1H), 1.68 (s, 3H), 1.55 – 1.09 (m, 9H), 1.04 – 0.78 (m, 12H); **^{13}C NMR** (101 MHz, $CDCl_3$) δ 132.16, 132.11, 68.16, 44.65, 43.92, 38.36, 32.07, 28.61, 26.86, 20.69, 19.54, 19.05, 18.99, 13.40, 12.83; **HRMS**-(ESI+) for $C_{15}H_{31}O$ $[M + H]^+$ calculated 227.2369 Da, found 227.2369 Da.

(2*E*,4*E*,6*S*,8*S*,10*S*)-Ethyl 4,6,8,10-tetramethyltrideca-2,4-dienoate (19):

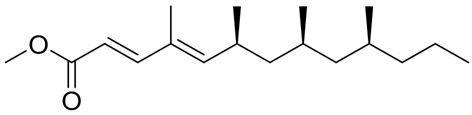

To a stirred solution of **17** (11 mg, 0.049 mmol) in DCM (1 mL) was added DMP (1.5 equiv, 0.16 mL, 0.073 mmol, 15 weight % solution in DCM). After 2 h, an aq saturated solution of $Na_2S_2O_3$ (5 mL) was added. Stirring was continued until both layers were clear and then the layers were separated. The aqueous layer was extracted with Et_2O (2 × 5 mL) and the combined organic fractions were dried ($MgSO_4$), filtered and concentrated. The crude aldehyde **18** was filtered over a short silica plug (pentane/Et_2O 10:1) and used in the next step without further purification.

To a stirred solution of triethyl phosphonoacetate (3 equiv, 24 μL, 0.12 mmol) in THF (0.8 mL) at 0 °C was added *n*-BuLi (2.2 equiv, 55 μL, 0.088 mmol, 1.6 M solution in hexanes). After 15 min, aldehyde **18** (9 mg, 0.040 mmol) was added and after completion (3–4 h), the reaction was quenched with aq saturated NH_4Cl (1 mL). The mixture was extracted with Et_2O (3 × 5 mL), dried ($MgSO_4$), filtered and concentrated. The crude product was purified using column chromatography (pentane/Et_2O 100:1) to afford pure **19** (7.5 mg, 64%) as a colorless oil.

^{1}H NMR (400 MHz, $CDCl_3$) δ 7.31 (d, *J* = 15.7, 1H), 5.78 (d, *J* = 15.7, 1H), 5.63 (d, *J* = 9.6, 1H), 4.21 (q, *J* = 7.0, 2H), 2.70 -2.59 (m, 1H), 1.79 (s, 3H), 1.50 – 1.43 (m, 1H), 1.43 – 1.17 (m, 9H), 1.18 – 1.10 (m, 1H), 1.09 – 0.99 (m, 2H), 0.96 (d, *J* = 6.6, 3H), 0.87 (t, *J* = 7.2, 3H), 0.81 (d, *J* = 5.4, 3H), 0.79 (d, *J* = 5.6, 3H); **^{13}C NMR** (101 MHz, $CDCl_3$) δ 167.64, 149.91, 148.63, 131.27, 115.48, 60.13, 45.69, 44.62, 39.33, 30.85, 29.62, 28.10, 21.22, 20.36, 20.04, 20.01, 14.39, 14.35, 12.34; **HRMS**-(ESI) for $C_{18}H_{33}O_2$ $[M + H]^+$ calculated 281.2475 Da, found 281.2473 Da.

(2*E*,4*E*,6*S*,8*S*,10*S*)-4,6,8,10-Tetramethyltrideca-2,4-dien-1-ol (2):

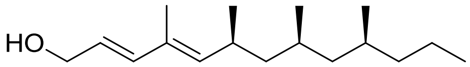

To a stirred solution of **19** (5 mg, 0.017 mmol) in DCM (1 mL) at −70 ºC was added DIBALH (3 equiv, 0.051 mmol, 1 M solution in DCM). After 1 h, TLC indicated complete consumption of the starting material, and the reaction was quenched with an aq saturated Rochelle salt (2 mL). The mixture was stirred until both layers were clear, and the product was extracted with Et_2O (3 × 5 mL). The combined organic layers were dried ($MgSO_4$), filtered and concentrated to afford **2** (4.1 mg, 96%) as a light-yellow oil.

^{1}H NMR (400 MHz, $CDCl_3$) δ 6.26 (d, *J* = 15.6, 1H), 5.72 (dt, *J* = 15.6, 6.2, 1H), 5.22 (d, *J* = 9.6, 1H), 4.20 (dd, *J* = 6.2, 1.0, 2H), 2.64 – 2.55 (m, 1H), 1.77 (s, 3H), 1.64 – 1.55 (m, 1H), 1.42 – 1.25 (m, 7H), 1.04 – 0.96 (m, 2H), 0.93 (d, *J* = 6.6, 3H), 0.91 – 0.84 (t, 3H, *J* = 7.2), 0.81 (d, *J* = 6.5, 3H), 0.80 (d, *J* = 6.6, 3H); **HRMS**-(ESI+) for $C_{17}H_{33}O$ $[M + H]^+$ calculated 253.2526 Da, found 253.2526 Da. $[\alpha]_D$= +7.1 (c = 0.1, pentane).

References

1. Matcha, K.; Madduri, A. V. R.; Roy, S.; Ziegler, S.; Waldmann, H.; Hirsch A. K. H.; Minnaard, A. J. *ChemBioChem* **2012**, *13*, 2537–2548.

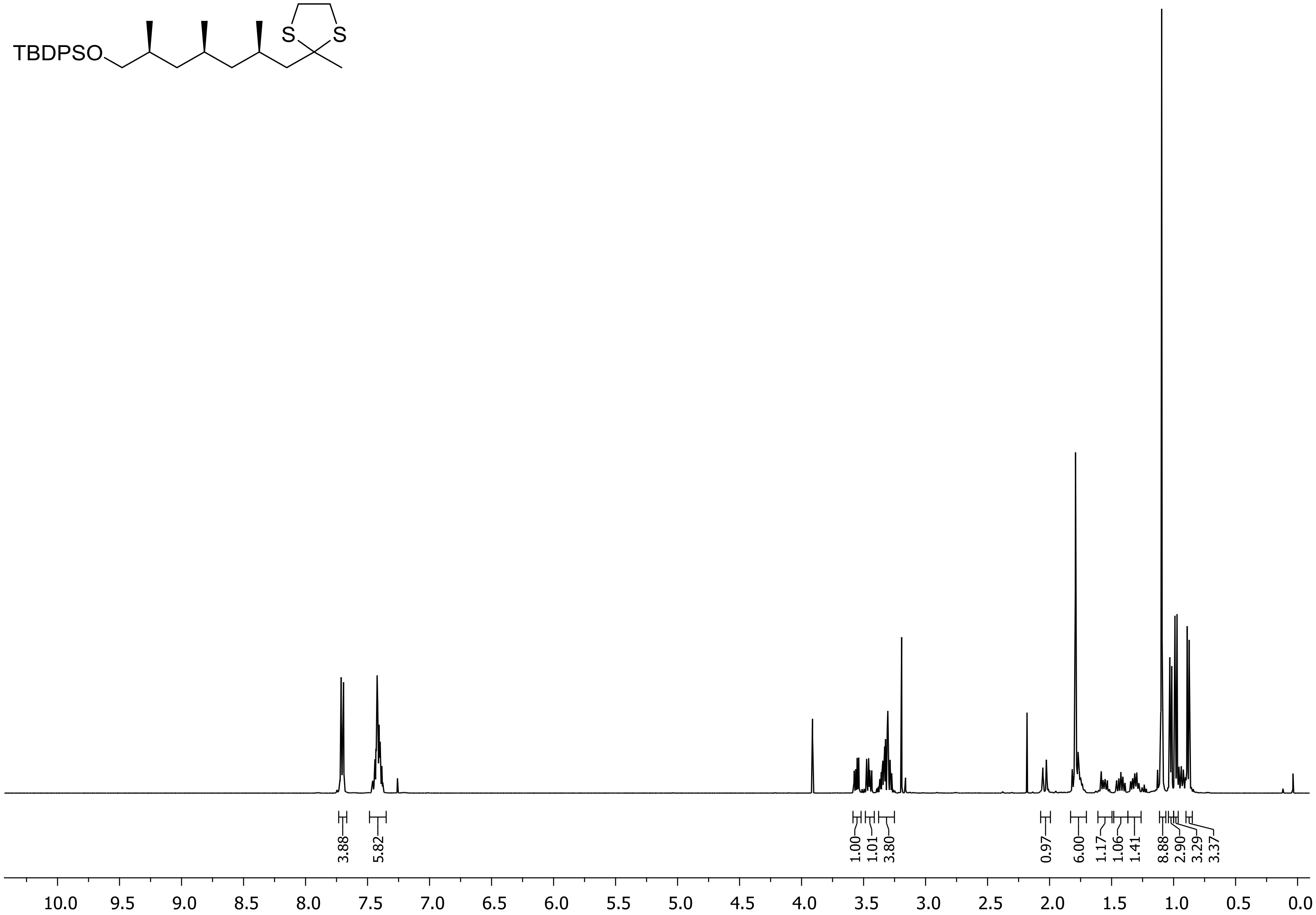
TBDPSO
S
S
3.88
5.82
1.00
1.01
3.80
0.97
6.00
1.17
1.06
1.41
8.88
2.90
3.29
3.37
10.0 9.5 9.0 8.5 8.0 7.5 7.0 6.5 6.0 5.5 5.0 4.5 4.0 3.5 3.0 2.5 2.0 1.5 1.0 0.5 0.0

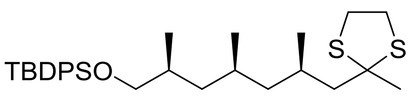
TBDPSO
S
S

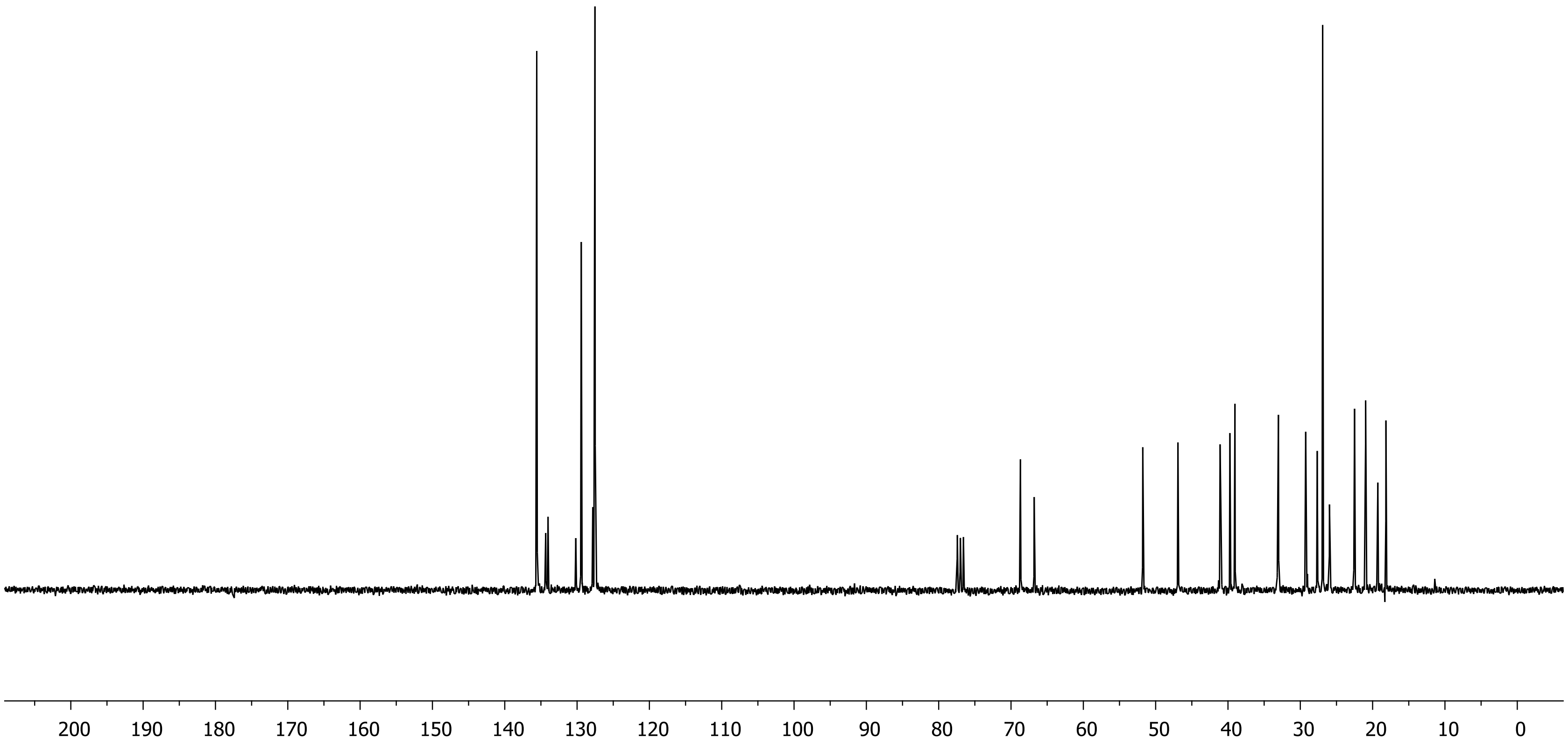
200 190 180 170 160 150 140 130 120 110 100 90 80 70 60 50 40 30 20 10 0

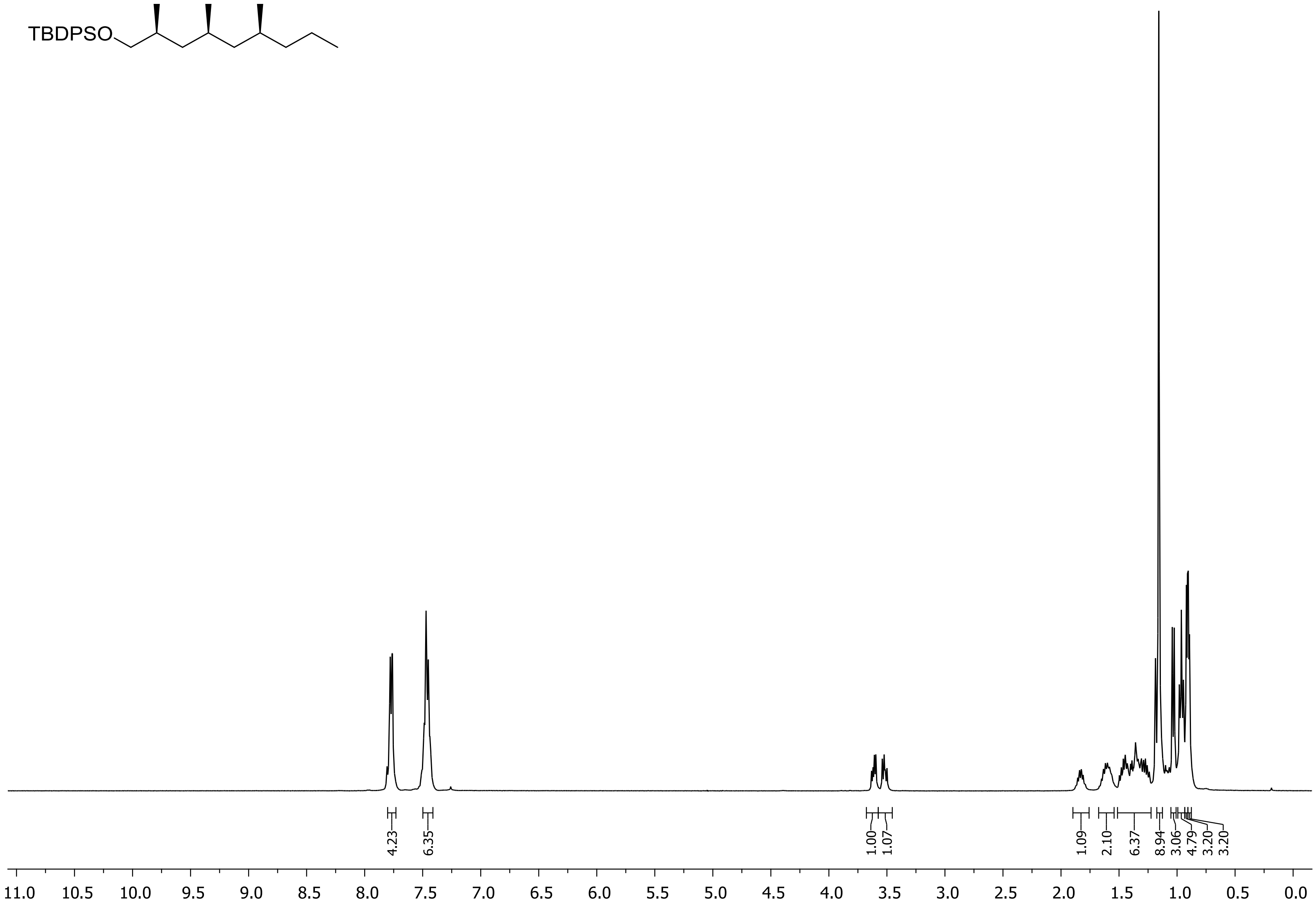
TBDPSO
4.23
6.35
1.00
1.07
1.09
2.10
6.37
8.94
3.06
4.79
3.20
3.20
11.0 10.5 10.0 9.5 9.0 8.5 8.0 7.5 7.0 6.5 6.0 5.5 5.0 4.5 4.0 3.5 3.0 2.5 2.0 1.5 1.0 0.5 0.0

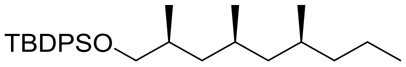
TBDPSO

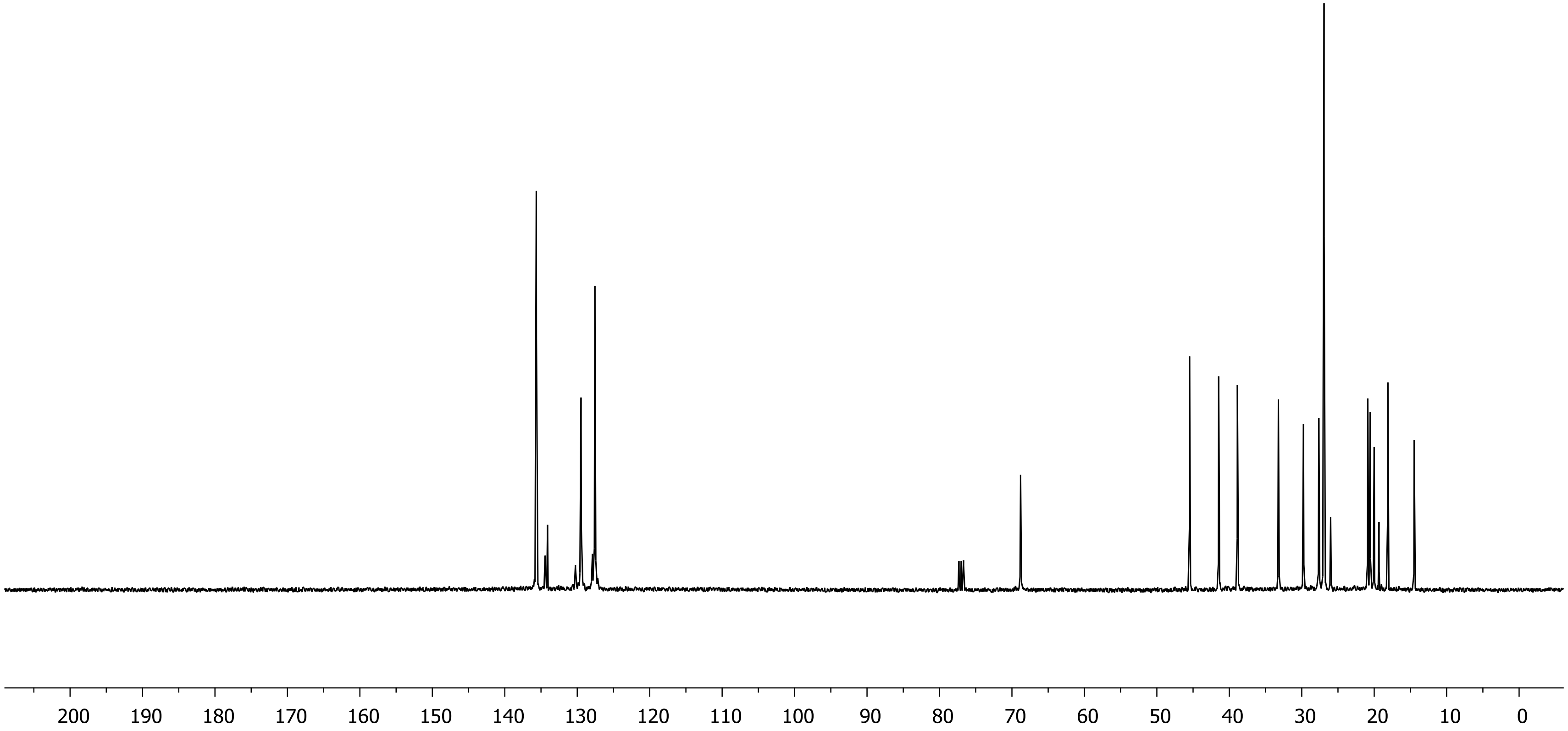
200
190
180
170
160
150
140
130
120
110
100
90
80
70
60
50
40
30
20
10
0

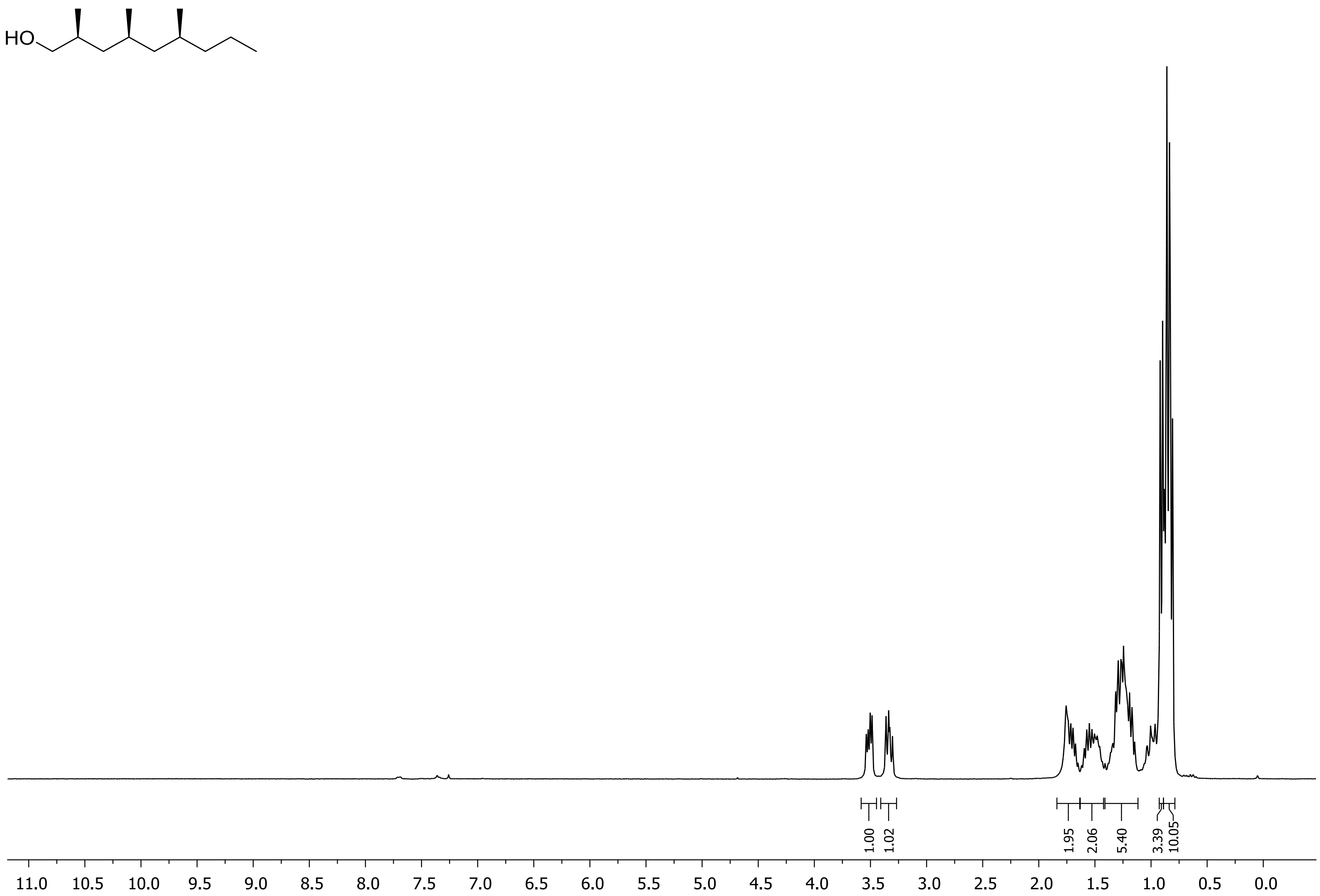
HO
1.00
1.02
1.95
2.06
5.40
3.39
10.05
11.0 10.5 10.0 9.5 9.0 8.5 8.0 7.5 7.0 6.5 6.0 5.5 5.0 4.5 4.0 3.5 3.0 2.5 2.0 1.5 1.0 0.5 0.0

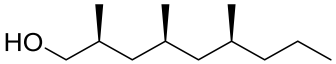

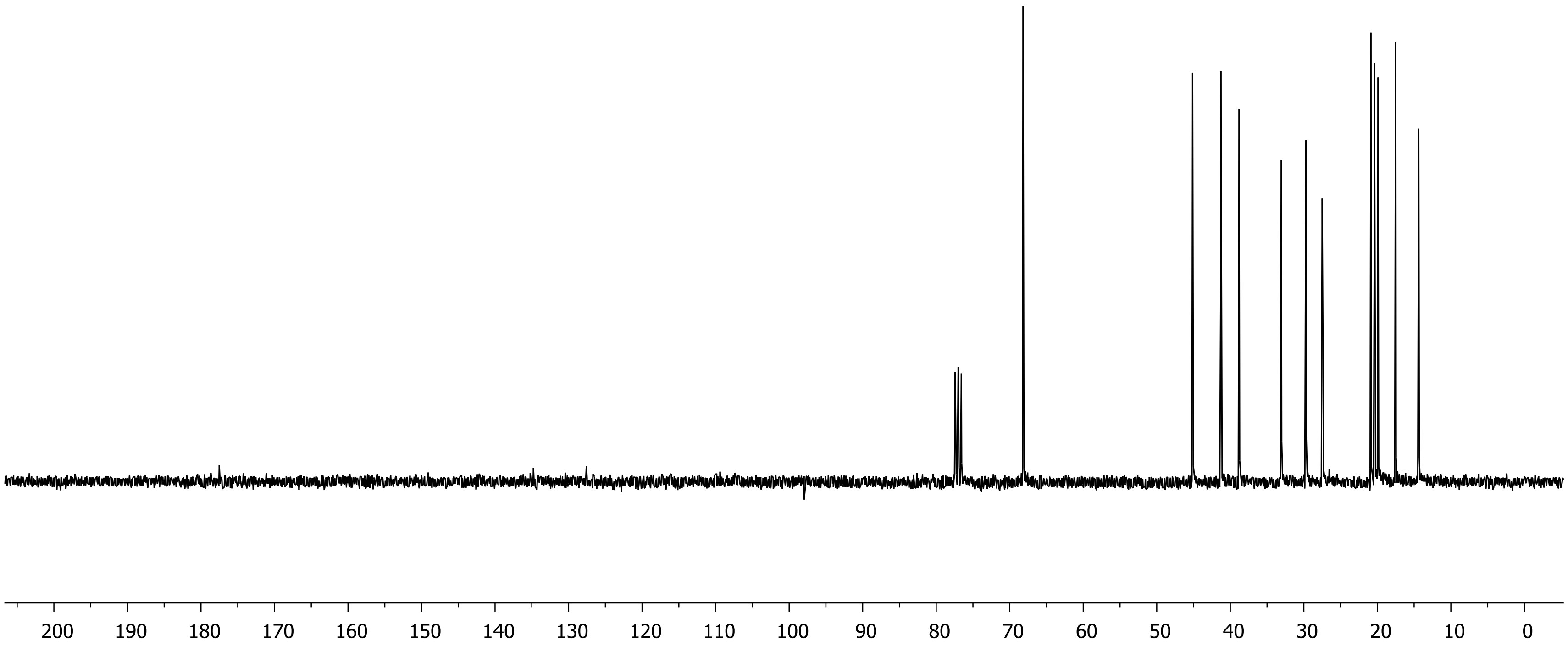

200
190
180
170
160
150
140
130
120
110
100
90
80
70
60
50
40
30
20
10
0

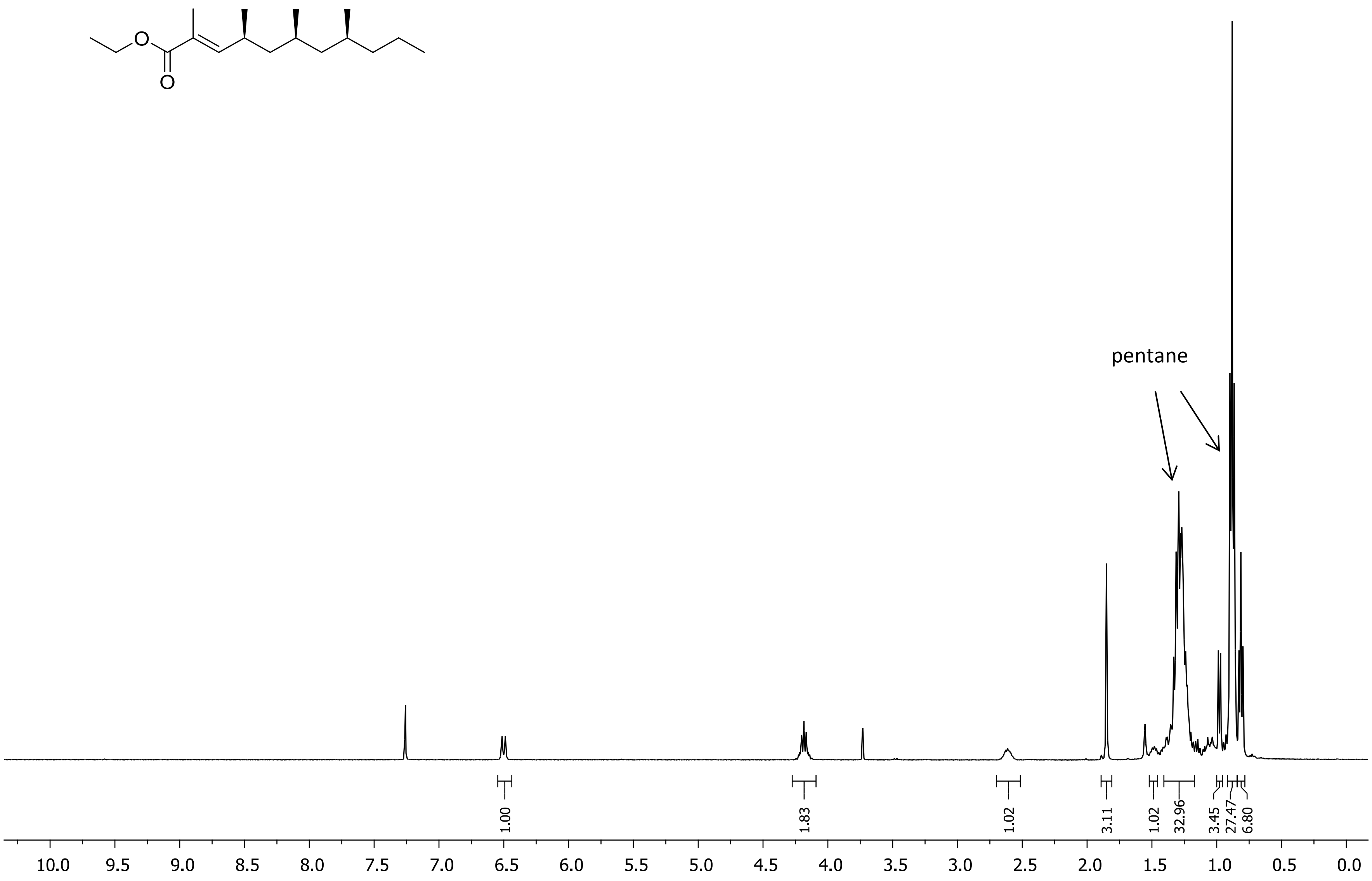
O
O
pentane
1.00
1.83
1.02
3.11
1.02
32.96
3.45
27.47
6.80
10.0
9.5
9.0
8.5
8.0
7.5
7.0
6.5
6.0
5.5
5.0
4.5
4.0
3.5
3.0
2.5
2.0
1.5
1.0
0.5
0.0

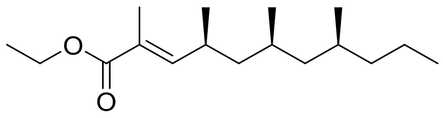
O
O

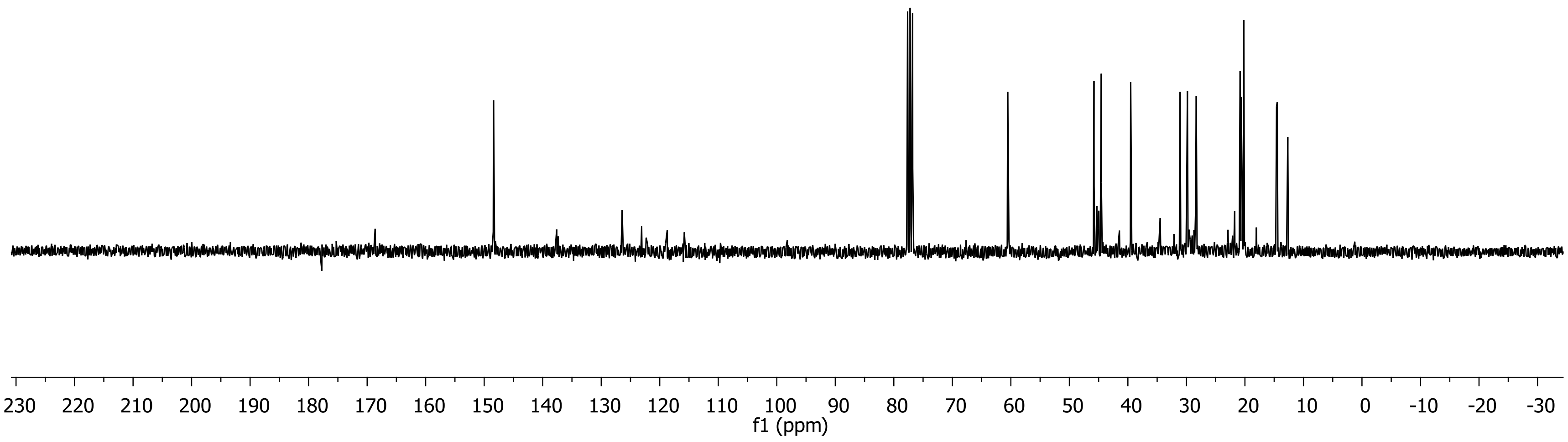
230 220 210 200 190 180 170 160 150 140 130 120 110 100 90 80 70 60 50 40 30 20 10 0 -10 -20 -30
f1 (ppm)

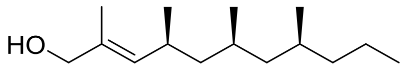
HO

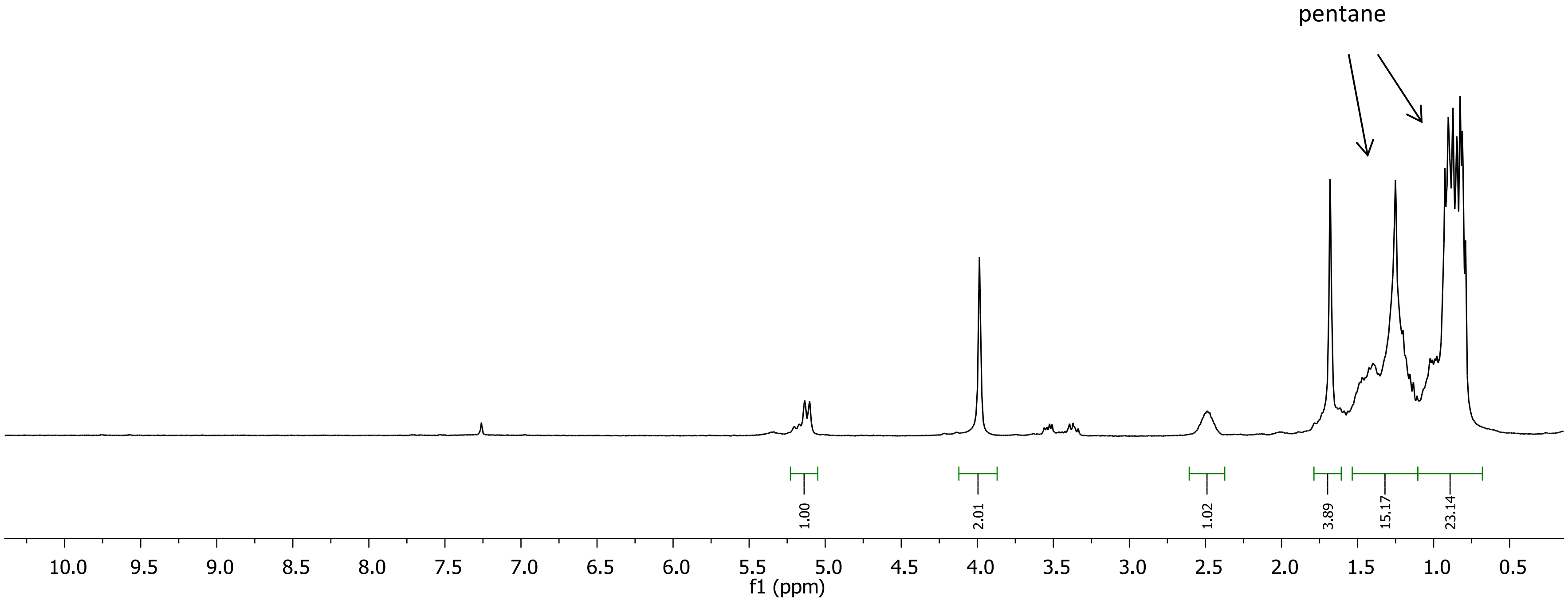
pentane
1.00
2.01
1.02
3.89
15.17
23.14
10.0 9.5 9.0 8.5 8.0 7.5 7.0 6.5 6.0 5.5 5.0 4.5 4.0 3.5 3.0 2.5 2.0 1.5 1.0 0.5
f1 (ppm)

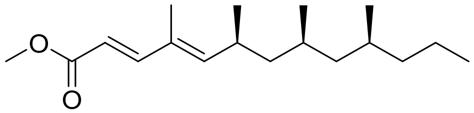

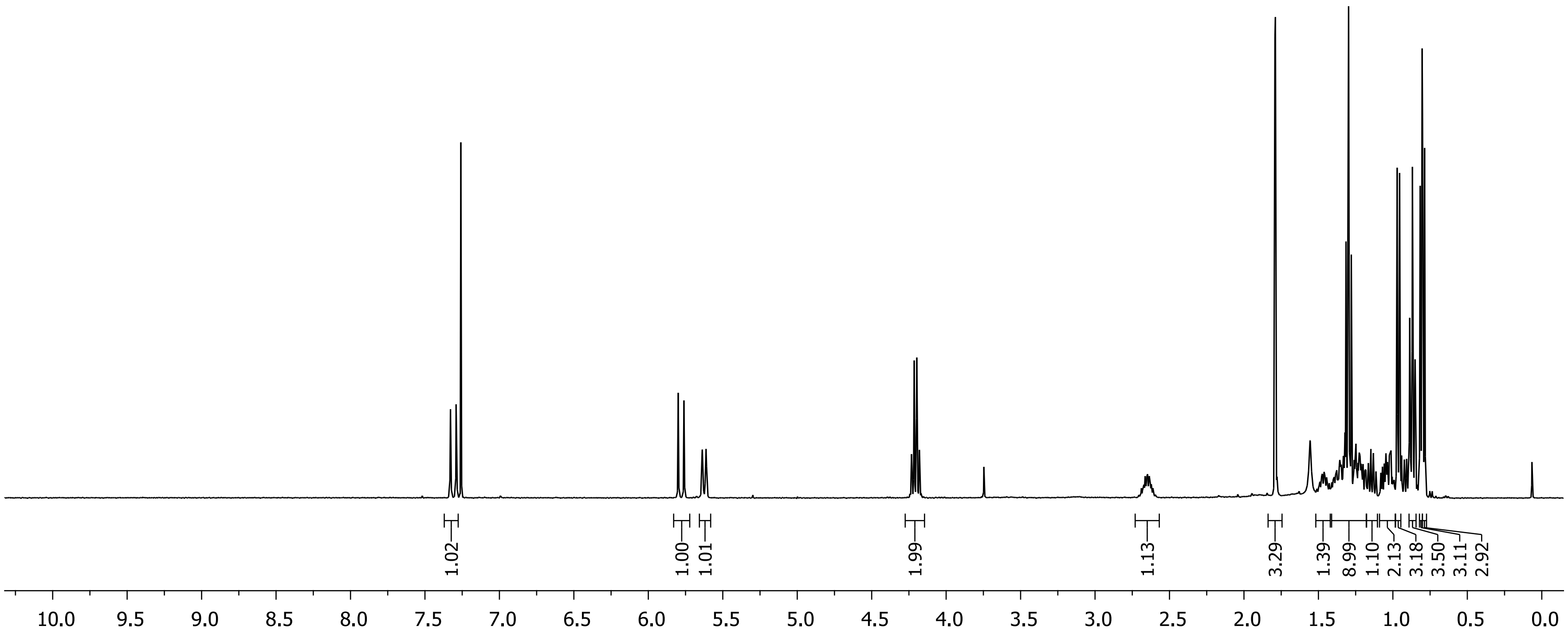
1.02
1.00
1.01
1.99
1.13
3.29
1.39
8.99
1.10
2.13
3.18
3.50
3.11
2.92
10.0 9.5 9.0 8.5 8.0 7.5 7.0 6.5 6.0 5.5 5.0 4.5 4.0 3.5 3.0 2.5 2.0 1.5 1.0 0.5 0.0

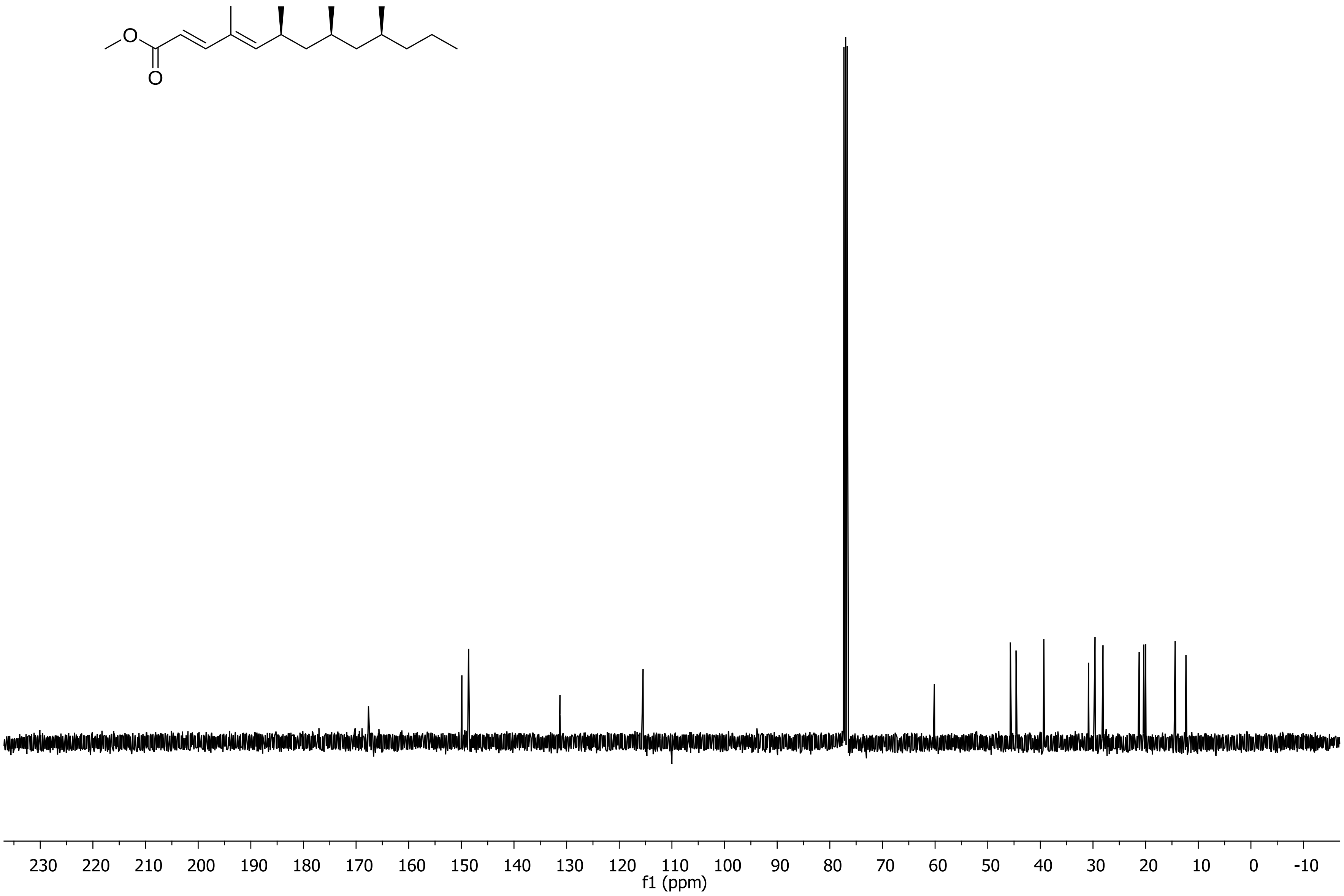
O
O
230 220 210 200 190 180 170 160 150 140 130 120 110 100 90 80 70 60 50 40 30 20 10 0 -10
f1 (ppm)

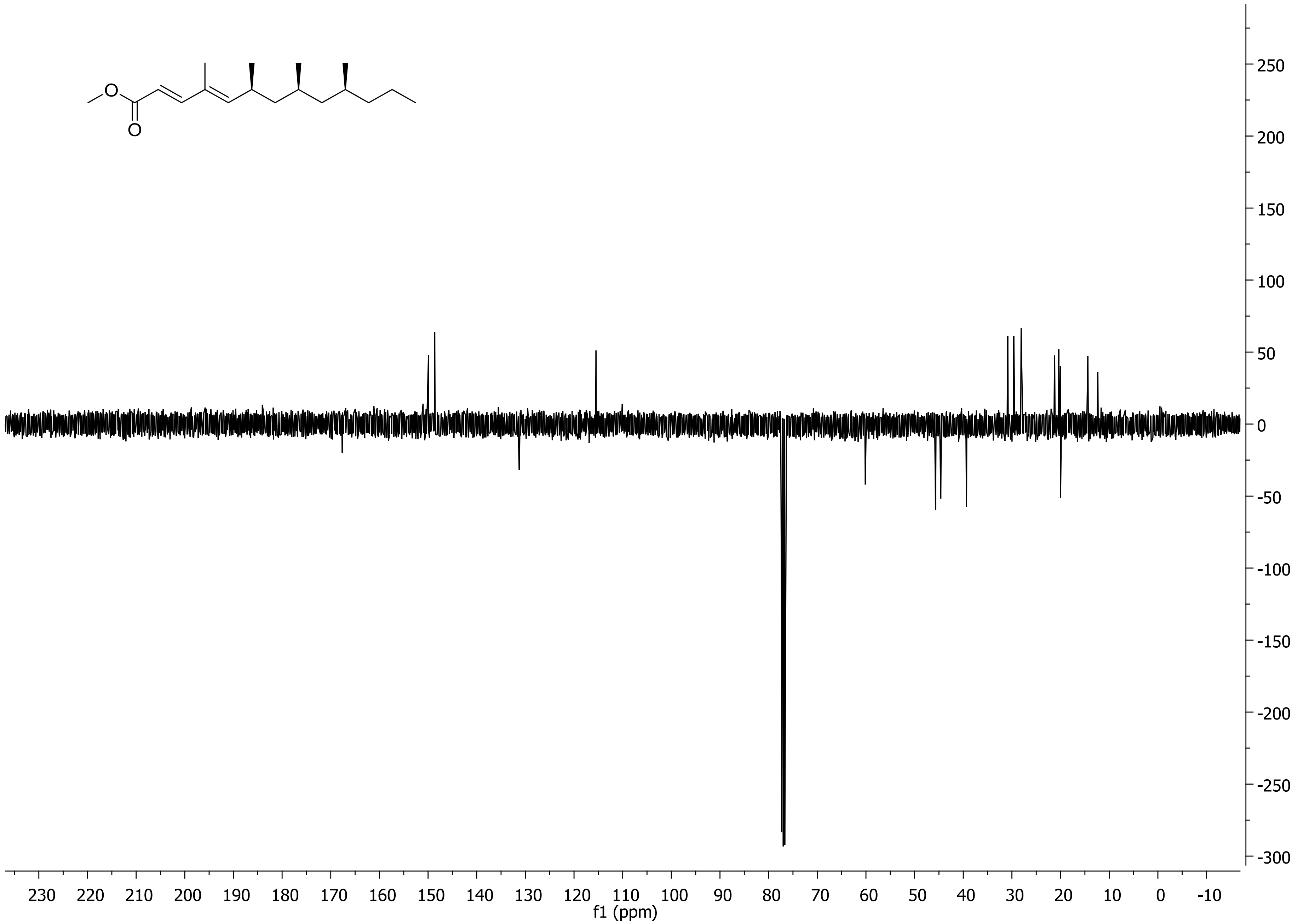
250
200
150
100
50
0
-50
-100
-150
-200
-250
-300
230 220 210 200 190 180 170 160 150 140 130 120 110 100 90 80 70 60 50 40 30 20 10 0 -10
f1 (ppm)

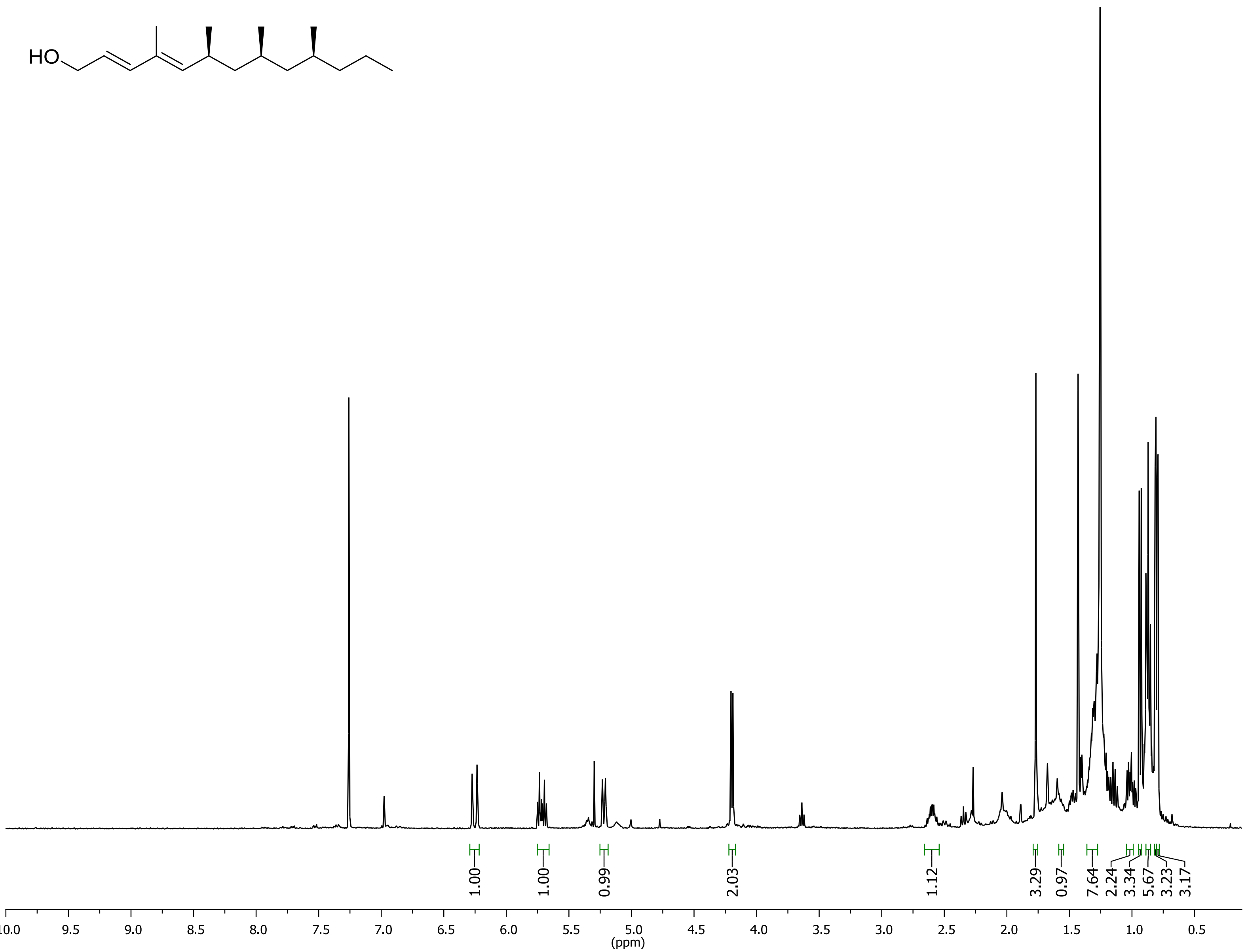
HO
1.00
1.00
0.99
2.03
1.12
3.29
0.97
7.64
2.24
3.34
5.67
3.23
3.17
10.0
9.5
9.0
8.5
8.0
7.5
7.0
6.5
6.0
5.5
5.0
(ppm)
4.5
4.0
3.5
3.0
2.5
2.0
1.5
1.0
0.5
